# Antibiotic Sales in Primary Care in Hubei Province, China: An Analysis of 2012–2017 Procurement Records

**DOI:** 10.3390/ijerph16183376

**Published:** 2019-09-12

**Authors:** Xinping Zhang, Youwen Cui, Chaojie Liu, Keyuan Zuo, Yuqing Tang

**Affiliations:** 1 School of Medicine and Health Management, Tongji Medical College, Huazhong University of Science and Technology, Wuhan 430030, Chinayouwencui2018@163.com (Y.C.); 2 School of Psychology and Public Health, La Trobe University, Melbourne, VIC 3086, Australia; C.Liu@latrobe.edu.au; 3 Department of Pharmaceuticals Bidding and Procurement, Hubei Public Resource Trading Center, Wuhan 430030, China; dr_zky@163.com

**Keywords:** primary care, antibiotic sales, China, quality indicators

## Abstract

The over-use of antibiotics has been identified as a major global challenge, where there is insufficient knowledge about the use of antibiotics in primary healthcare settings, especially at a population level. This study aims to investigate the trends and patterns of antibiotic sales in primary care in Hubei, China over a six-year period from 2012 to 2017. Antibiotic sales were expressed with Defined Daily Doses per 1000 inhabitants per day (DIDs) and compared with European countries using the 12 quality indicators proposed by the scientific advisory board of the European Surveillance of Antimicrobial Consumption (ESAC) project. Antibiotic sales increased from 12.8 DID in 2012 to 15.3 DID in 2013, and then declined afterwards. The most commonly used antibiotics, J01C (beta-lactam antimicrobials, penicillins), accounted for 40.5% of total antibiotic sales. Parenteral administration of antibiotics accounted for over 50% of total antibiotic sales. Total antibiotic sales were almost on a par with the 31 European countries monitored by the ESAC project, but cephalosporin sales were higher than at least three quarters of the compared countries, resulting in a significant higher proportion of third-generation cephalosporin consumption (13.8–19.43%). The relative consumption of Fluoroquinolone (9.26–9.89%) was also higher than at least half of the compared countries. There is a lack of robust evidence to show that antibiotic consumption in primary care is lower in Hubei compared with other countries. The preference of clinicians in China to use broad-spectrum and parenteral antibiotics deserves further study and policy attention.

## 1. Introduction

The over-use of antibiotics has been identified as a major global challenge, especially in low- and middle-income countries (LMIC) [1,2]. It has been proved to be associated with the development and spread of antimicrobial resistance (AMR), an alarming global public health threat. Deaths attributed to drug-resistant infections may surpass 10 million in 2050, and could result in an estimated $US100 trillion loss in global economic output if the rising trend is not properly contained from the current level of 700,000 deaths annually [3]. There is also an urgent global need to find new antibiotics [4].

Global consumption of antibiotics increased by 39% between 2000 and 2015 [5], with the majority being dispensed from primary care facilities [6]. Irrational antibiotic prescriptions are most prevalent in the primary care sector [7,8]. China is no exception [9]. The primary care network in China, including community/township health centres and their outreach stations/clinics [10], provided 54.2% of outpatient care (4.43 billion visits) and 18.2% of inpatient care (44.5 million hospital admissions) in 2017 [11]. The over-prescription of antibiotics is common in these facilities [12]. In Shandong, for example, primary care institutions dispensed about 80% of all antibiotics for medical care. Previous studies have proven that the irrational use of antibiotics in primary care, especially for longer and more frequent antibiotic courses, contributes to the asymptomatic carriage of resistant bacteria, which can last for up to 12 months [13,14,15,16].

Efforts in many countries to contain antibiotic consumption in primary care have failed [17]. In China, it is widely believed that the over-prescription of medicines, including antibiotics, has its roots in the distorted pricing system introduced over the period when China transitioned from a planned economy to a market one in the 1980s. Inadequate government funding, fee for-service payments, and the 15% profit margin on the sales of medicines incentivised over-prescription. Antibiotics became one of the most commonly abused pharmaceutical products [18,19]. Despite policy interventions imposing a cap on the sales of medicines as a percentage of total revenue, over-prescription was still deemed a big problem [20,21,22]. It was estimated that the sales of medicines accounted for 44.0% of hospital revenue and 59.1% of primary care revenue, respectively, in 2008 [23].

Attempting to further curtail irrational prescriptions in primary care, the Chinese government launched a round of comprehensive system reforms in 2009. Prescriptions in primary care were restricted to those included on the essential medicines list (EML). A profit margin for the sales of medicines was disallowed. A regional government tendering platform was set up to procure essential medicines for primary care facilities [24]. Empirical data showed limited evidence of the policy impact on antibiotic prescribing practices, despite a drop in the price of essential medicines [25,26,27].

In 2012, the Chinese government issued new “*administrative rules for the clinical use of antibiotics*”. This is considered the most rigid regulatory control over antibiotic prescriptions to date [28,29]. Antibiotics were categorised into three groups: Non-restricted, restricted and controlled; and controlled antibiotics were excluded from the EML for primary care. Prescriptions of restricted (or controlled) antibiotics were subject to strict administrative restrictions. Penalties are applied for violating the rules [30].

Little is known of the effects of the 2012 policy. This study aimed to investigate the trends and patterns of antibiotic sales in primary care in Hubei over a six-year period from 2012 to 2017, in comparison with the European Surveillance of Antimicrobial Consumption (ESAC) results [31].

## 2. Materials and Methods

This study analysed the aggregated data of antibiotic procurement for all public primary care facilities in Hubei, China over the period from 2012 to 2017. Approximately 60% of outpatient visits in 2017 in Hubei province were in primary care facilities (Table 1). Public primary care institutions in Hubei share approximately 75% of patient visits to all primary care facilities [32]. The public primary care facilities thus shared approximately 45% (60% multiplied by 75%) of total outpatient visits in Hubei province in 2017.

### 2.1. Study Setting

Hubei province is located in central China, with a population of over 59 million (in 2017) across a geographic area of 185,900 km^2^. The annual average income per capita in Hubei ranks in the middle range of all provinces. In 2016, urban residents in Hubei had an average annual income of 12,725 Yuan (US$1916) per person compared with 12,363 Yuan (US$1861) across the country, while rural residents had an average annual income of 29,386 Yuan (US$4424) per person compared with 33,616 Yuan (US$5061) across the country [33]. About 5.89% of the gross domestic product (GDP) in Hubei was spent on health. In 2017, Hubei had 2.50 physicians, 3.12 nurses, and 6.37 beds per 1000 residents in the health care sector [34].

Hubei started to introduce the essential medicines system in 2010. Universal coverage was achieved the next year. In line with the national strategy, the “*administrative rules for the clinical use of antibiotics*” has been implemented since August 2012. In the “*management measures for the clinical use of essential medicines*” issued in November 2014, the Hubei government set a clear target to bring antibiotic prescriptions in primary health care settings down to no more than 20% for outpatient prescriptions and 40% for prescriptions in emergency care, respectively. The clinical use of antibiotics was capped at 40 defined daily doses (DDD) per hundred patient days [35].

### 2.2. Data Source

Data for this study were extracted from the Hubei Medical Procurement Administrative System (HMPAS). The HMPAS is the only online platform run by the government to procure pharmaceutical products for public facilities in Hubei. It was introduced in 2011 for public-owned primary care facilities.

Public primary care institutions can only order medicines from the HMPAS on a monthly basis. The system records the volume and expense of each type of medicine purchased by each facility. The procured medicines are categorised using the anatomical therapeutic chemical (ATC) classification codes. Records of “J01” (antibacterial for systemic use) procurement for primary care facilities from 2012 to 2017 were extracted for the purpose of this study.

### 2.3. Data Analysis

Antibiotic sales were measured using the DDD method developed by the World Health Organisation (WHO) [36]. DDD indicates the average maintenance dose per day for a drug used for its main indication in adults. According to the WHO Collaborative Centre for Drug Statistics Methodology, DDD equivalence per package (DPP) of medicines was calculated using the formula: DPP = (unit strength × pack size/DDD). The total volume of procurement was estimated as the summed DDDs of all packaged products.

(1) $$DDDs=\sum_{i=1}^{n}\left(DPP_{i}\times N_{i}\right)$$

where *N_i_* represents the number of packages of certain product (*i*).

We calculated the DDDs for systemic antibiotics (J01) and its subsequent subgroups such as (J01C) penicillin and (J01D) cephalosporin (a full list of subgroups can be found in Table 2). The DDDs were then transformed into DDDs per 1000 inhabitants per day (DIDs) in order to determine the antibiotic sales trends over the years. The number of inhabitants was denoted as end-of-the-year population size in the annual statistical reports. Since 25% of patient visits to primary care facilities are not covered by the public primary care institutions, we reduced the denominator (end of the year population size) by 25% with the assumption that the majority of patients visiting private institutions generally do not go to public institutions and consultation rates would be similar in both groups.

The 12 quality indicators measuring antibiotic use in primary care proposed by the scientific advisory board of the European Surveillance of Antimicrobial Consumption (ESAC) project were adopted to assess the potential irrational use of antibiotics [31]. The 12 quality indicators cover DIDs of J01 and its four subgroups J01C (beta-lactam antimicrobials, penicillins), J01D (other beta-lactam antimicrobials), J01F (macrolides, lincosamides and streptogramins) and J01M (quinolone antibacterial), as well as the percentages of J01CE (beta-lactamase sensitive penicillins), J01CR (combinations of penicillins, incl. beta-lactamase inhibitors), J01DD (third-generation cephalosporins) + DE (fourth-generation cephalosporins), and J01MA (fluoroquinolones) in total antibiotic (J01) sales. The pattern of antibiotic sales was also measured by the ratio of broad and narrow spectrum antibiotics and seasonal variations of J01 and J01M sales. The ESAC published the performance of 31 European countries on the 12 quality indicators from 2012 to 2017 for benchmarking [37]. In the benchmarking analysis, we adjusted the absolute antibiotic sales figures in line with the market share of public primary care facilities in outpatient visits in Hubei province in 2017 (45% in Hubei province). The estimated outpatient antibiotics sales were then compared with 31 European countries in the corresponding year from 2012 to 2017. This is not perfect, as in European countries some small proportion of outpatients are seen in the hospital, while our adjustment assumes this is zero.

## 3. Results

**Figure 1:**
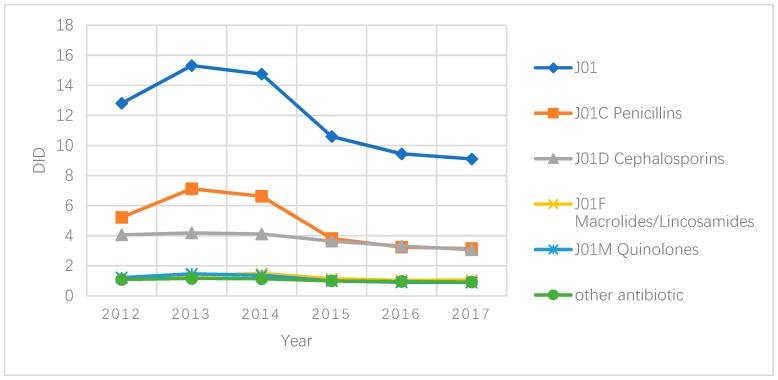
Antibiotic sales (DID) in primary care settings in Hubei, China, 2012–2017.

On average, antibiotic sales in primary care in Hubei increased from 12.8 DID in 2012 to 15.3 DID in 2013, and then declined afterwards. The year of 2015 experienced the sharpest drop, down from 14.8 DID to 10.5 DID. This was followed by a slight further decline to 9.5 DID in 2016 and 9.1 DID in 2017. Such a trend was mainly shaped by changes in penicillin sales. The decrease in the sales of other antibiotic products was much less dramatic (Figure 1).

The most commonly used antibiotics were J01C (beta-lactam antimicrobials, penicillins), accounting for 40.5% (ranging from 34.3% to 46.5% over the years) of total antibiotic sales, followed by J01D (other beta-lactam antimicrobials; 31.1%), J01F (macrolides, lincosamides and streptogramins; 10.2%) and J01M (quinolone antibacterial; 9.5%). But since 2015, the sales of cephalosporins have been on par with that of penicillins.

**Figure 2:**
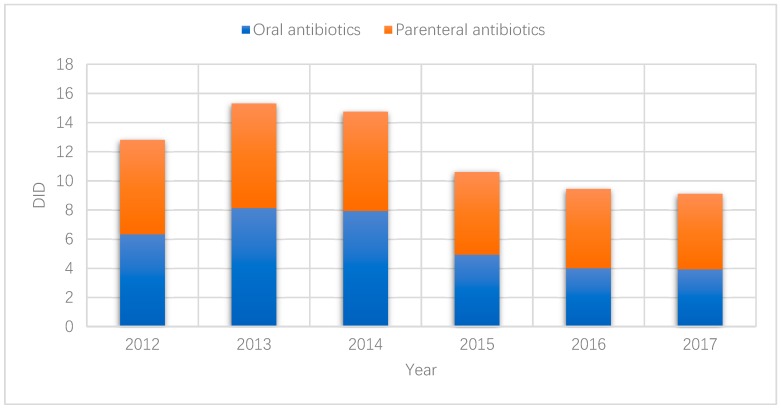
Sales of oral and parenteral antibiotics in primary care settings in Hubei, China, 2012–2017.

The percentage of parenteral administration of antibiotics experienced a slight increase over the years, up from about 50% to over 55% (Figure 2).

Extended-spectrum penicillins (J01CA) dominated the sales of penicillins, accounting for 73.5% of the total antibiotic sales (ranging from 66.6% to 78.1% over the years). Although the sales of both extended-spectrum (from the peak of 5.388 DID in 2013 to 2.095 DID in 2017) and narrow-spectrum penicillins (from 1.281DID in 2013 to 0.376 DID in 2017) dropped, the use of combinations of penicillins increased steadily from 0.358 DID in 2012 to 0.676 DID in 2017 (Table 2).

The sales of first, second and third generations of cephalosporin contributed to 16.0% (ranging from 10.84% to 19.77% over the years), 31.1% (ranging from 23.46% to 36.19% over the years) and 53.0% (ranging from 49.34% to 57.41% over the years) of the total sales of cephalosporin (J01D). Overall, cephalosporin sales declined over the years across all of the three generations (Table 2).

Fluoroquinolone (J01MA) was the only quinolone product used in primary care in Hubei. Its sales have decreased steadily over the years. Macrolides and lincosamides accounted for 63.3% and 36.7% of the total J01F (macrolides, lincosamides and streptogramins) sales, respectively. Aminoglycoside antibacterials, sulfonamides and trimethoprim and imidazole derivatives accounted for 1.1%, 0.1% and 6.1% of the total antibiotic sales, respectively. Similarly, their sales also declined over the years.

Overall, estimated outpatient antibiotic sales in Hubei were almost on a par with the 31 European countries monitored by the ESAC project (Table 3). Penicillin (J01C) sales were low after 2016, not only in absolute terms (DID) but also in relative terms as a percentage of total antibiotic sales. The sales of J01F (macrolides, lincosamides and streptogramins) and J01M (quinolone antibacterial) were in the low/middle or high range of the compared countries. But cephalosporin (J01D) sales were higher than at least three quarters of the compared countries, resulting in a significant higher proportion of J01DD (third-generation cephalosporin) sales (13.8–19.43%) compared with the 31 European countries (8.4–8.9%). The relative sales of J01MA (Fluoroquinolone, 9.26–9.89%) and J01CE (beta-lactamase sensitive penicillins, 3.8–3.72%) as a percentage of total antibiotic sales were also higher than at least half of the compared countries. The ratio of broad/narrow spectrum antibiotics increased over the years, but still fell into the lower end of the compared countries. Seasonal variations in both total antibiotic and quinolone sales were lower than most of the compared countries (Table 3).

## 4. Discussion

To the best of our knowledge, this is the first study of its kind to estimate population-wide antibiotic use in primary care in central China, using the widely accepted quality indicators (QIs) proposed by the ESAC project. Through a benchmarking analysis, we revealed some patterns of antibiotic use in primary care that were unknown to previous studies.

The total antibiotic sales in public primary care facilities in Hubei as expressed by DID are similar or even lower compared with those reported by the ESAC study countries. This is unexpected since previous studies illustrated a prescribing pattern in China characterised by the over-prescription of antibiotics. This finding should be interpreted with caution. Public primary care facilities share about 45% of the total volume of outpatient visits in Hubei, much lower in comparison with the European countries. In most European countries, more than 80% of outpatient encounters occur in primary care facilities [38]. In the absence of a “gate-keeping” role of primary care services, patients enjoy the freedom to choose hospitals as their preferred first contacts. Empirical evidence shows that consumer demands for non-referred outpatient services in hospitals have always been high in China [39]. There is a great chance that many patients may bypass primary care and seek more expensive outpatient care from hospitals [40]. In 2017, for example, on average, each Hubei resident made 5.77 outpatient visits: 60% in primary care facilities (75% shared by public facilities) and 40% in hospitals. (Table 1). A study found that hospital consumption of antibiotics actually increased over the period from 2011 to 2014 in China [41]. The low antibiotic sales in primary care in Hubei may simply be a reflection of low consumer demands for primary care. It would be higher than the ESAC countries if the share of outpatient visits in primary care increased from the current level of 45% to a level aligned with the ESAC countries. This is likely to be the case given that previous studies revealed consistently high levels of antibiotic prescription in primary care in China, for example, 60% for upper respiratory infections [42]. We adjusted the absolute antibiotic sales figures in line with the market share of primary care facilities in outpatient visits. The results indicate that the sale of J01D in primary care in Hubei was higher than in most of the European countries.

However, the adjustment method used in this study has some limitations by itself. For example, antibiotic prescription rates vary across the primary care and hospital sectors due to differences in patient profiles. Over-the-counter (OTC) sales of antibiotics are another serious problem in China. A recent study revealed that more than 55% of community pharmacies in China sell antibiotics upon requests from their clients without requiring a prescription [43]. A global study showed that the weighted non-prescription use of antibiotics in China is around 36%, compared with 6% in central Europe, 19% in southern Europe, and 3% in northern Europe [44]. OTC sales of antibiotics would reduce the need of antibiotic prescriptions.

Accessibility to healthcare can also influence sales of antibiotics in healthcare facilities. China has achieved almost universal (>96%) coverage of health insurance. Governmental subsidies to primary care institutions increased from ¥19 billion (US$2.8 billion) in 2008 to ¥140 billion ($20.3 billion) in 2015 [10]. The 2013 National Health Services Surveys show that over 85% of people in China visited a doctor when needed [45]. The universal health coverage index in China reached 76 according to the World Health Statics 2018, on par with the average level of European countries (>80 in 14 European countries; 54–79 in 36 European countries).

In saying this, however, there is evidence that antibiotic sales are declining in primary care. This study revealed that total antibiotic sales in public primary care facilities in Hubei decreased more rapidly than the decline in patient visits, if any, over the study period. Several policy initiatives may have contributed to these results. Previous experience in China indicates that persuasive measures to reduce the overuse of antibiotics had only scant success. For example, the 2004 national guidelines on antibiotic use were criticised for its fragmented and incomplete approach [46]. Several restrictive measures imposed in the series of national essential medicines policies since 2009, such as the limited availability of antibiotics and zero-markup for sales of medicines in primary care, also had a limited effect on antibiotic prescribing practices [19,20,21]. This triggered stronger administrative interventions. The 2012 “*Administrative Rules for the Clinical Use of Antibiotics*” issued by the Hubei government appeared to work better according to findings of this study and others [47]. The dramatic decline in antibiotic sales in 2015, as shown in this study, also coincided with the “*Management Measures on the Use of Essential Medicines in Medical Institutions in Hubei Province*”. For the first time, the Hubei government set a clear target to curb antibiotic use. In addition to persuasive guidelines, penalties applied for violating these administrative rules and measures. These included downgraded accreditation and service fee levels and the dismissal of managers. Medical workers involved may lose permission to prescribe antibiotics, or even have their medical registration revoked.

The quality use of antibiotics remains a concern in primary care in Hubei. This study found that most of the reduction in antibiotic sales occurred on two narrow-spectrum antibiotics, J01CE (beta-lactamase sensitive penicillins) and J01DB (first-generation cephalosporins), compared with limited reduction in broad-spectrum antibiotics such as J01DD (third-generation cephalosporins) and J01DC (second-generation cephalosporins). The sales of J01D (cephalosporins) in Hubei are significantly higher than those of most ESAC countries despite a lower volume of patient visits. Cephalosporins, together with broad-spectrum penicillins, are the most consumed antibiotics in the world, accounting for 55% of the total antibiotics consumed in 2010. Between 2000 and 2010, cephalosporins experienced a large increase in sales globally [1]. The preference of clinicians in China to use cephalosporin (including β-lactamase inhibitor combination preparations) and fluoroquinolones is deemed a serious problem of professional incompetence [48]. This study also found that third-generation cephalosporins, mostly second-line treatment options, accounted for more than half of the total cephalosporin sales. Such a pattern was also observed in some countries in Eastern Europe and Central Asia [49]. Overuse of third-generation cephalosporins could induce the emergence and spread of drug-resistant strains of microbes, such as the vancomycin-resistant enterococci (VRE), the ESBLs-producing *Klebsiella pneumoniae*, and multi-drug resistant Gram-negative bacteria [50,51].

The overuse of parenteral antibiotic treatment is another global concern. The percentage of parenteral antibiotic sales (50–55%) in Hubei as revealed in this study is much higher compared with European countries (ranging from 0.001% in Iceland to 6.75% in Russia) [52]. This result is consistent with findings of previous studies [53]. Although the sales of medicines generated a zero profit margin in primary care in China, a fee could be charged for administering injections or infusions [10]. Patient preference may further complicate the situation. In China, patients often perceive injections as being powerful, fast-acting, and longer lasting than oral pills, putting additional pressure on prescribers [54].

The national roadmap for health system development, Healthy China 2030, has highlighted the importance of primary care. With strong political will and commitment, the primary care sector in China is expected to be strengthened. Pilot trials in Shenzhen have demonstrated the steady growth in the share of patient visits in primary care when primary care providers serve as first point of contact in the integrated care delivery system [55]. In September 2017, the health ministry of China decided to extend this approach to the entire country. This will undoubtedly increase antibiotic sales in primary care. Increasing attention should be paid to the quality use of antibiotics in primary care, in particular, the potential excessive use of broad-spectrum antibiotics such as third-generation cephalosporins and the parenteral administration of medicines.

There are several limitations in this study. Data used in this study were drawn from procurement records, which do not directly reflect the actual use of medicines. We were also unable to evaluate the appropriateness of antibiotic prescribing practices. The HAMPS records did not capture the medicines in stock or expired or discarded medicines. Private primary care facilities were excluded in this study, although they accounted for 25% of patient visits in primary care [56]. The procurement records did not separate rural and urban sales, preventing us from exploring rural–urban differences. We were not able to perform risk adjustments in the benchmarking analysis due to the unavailability of relevant data.

## 5. Conclusions

Using the procurement records, for the first time, we estimated antibiotic sales in public primary care facilities in Hubei over the period from 2012 to 2017. Public facilities shared 75% of patient visits to primary care in Hubei, with the total sales of antibiotics ranging from 9.1 to 15.3 DID across the study period. There is a lack of robust evidence to show that antibiotic sales in primary care are lower in Hubei compared with other countries. Low sales of antibiotics may be a result of low needs for prescriptions and the low market share of primary care in outpatient visits. However, it is evident that the sales of antibiotics in public primary care facilities in Hubei are declining, in particular for narrow-spectrum antibiotics. However, the sales of broad-spectrum cephalosporins have remained high. In addition, third-generation cephalosporins accounted for more than half of the total cephalosporin sales. A high percentage (50–55%) of parenteral antibiotic sales in Hubei were also found in this study. The preference of clinicians in China to use broad-spectrum and parenteral antibiotics deserves further study and policy attention.

## Figures and Tables

**Table 1:** Outpatient visit data for primary care and hospital sector in Hubei province, 2012–2017.

Year	Year-End Population (10,000)	Primary Care Visit	Hospital Sector Visit	Share (%) of Primary Care
2012	5799	194,226,467	92,367,048	67.77
2013	5799	221,885,235	98,597,112	69.23
2014	5816	239,407,480	109,767,232	68.56
2015	5851.5	205,444,029	117,219,783	63.67
2016	5885	212,090,000	126,660,279	62.61
2017	5902	205,080,000	135,382,043	60.24

**Table 2:** Sales of various categories of antibiotics in primary care in Hubei China (2012–2017).

ATC Classification	Sales Measured in DID (%)						Overall %
	2012	2013	2014	2015	2016	2017	
J01A TETRACYCLINES							
J01AA Tetracyclines	-	-	-	0.024 (0.22)	0.029 (0.31)	0.025 (0.27)	0.1
J01C BETA-LACTAM ANTIBACTERIALS, PENICILLINS							
J01CA Penicillins with extended spectrum	4.076 (31.82)	5.388 (35.19)	4.919 (33.37)	2.773 (26.17)	2.197 (23.27)	2.095 (23.00)	29.8
J01CE Beta-lactamase sensitive penicillins	0.725 (5.66)	1.227 (8.01)	1.152 (7.81)	0.452 (4.27)	0.359 (3.80)	0.346 (3.79)	5.9
J01CF Beta-lactamase resistant penicillins	0.062 (0.49)	0.055 (0.36)	0.051 (0.35)	0.042 (0.40)	0.034 (0.37)	0.030 (0.34)	0.4
J01CR Combinations of penicillins, including beta-lactamase inhibitors	0.358 (2.80)	0.450 (2.94)	0.500 (3.39)	0.550 (5.20)	0.653 (6.92)	0.676 (7.42)	4.5
J01D OTHER BETA-LACTAM ANTIBACTERIALS							
J01DB First-generation cephalosporins	0.776 (6.06)	0.828 (5.41)	0.761 (5.16)	0.522 (4.93)	0.368 (3.90)	0.333 (3.66)	5.0
J01DC Second-generation cephalosporins	0.952 (7.43)	1.184 (7.73)	1.326 (9.00)	1.247 (11.76)	1.109 (11.73)	1.113 (12.23)	9.7
J01DD Third-generation cephalosporins	2.329 (18.18)	2.176 (14.21)	2.034 (13.79)	1.870 (17.65)	1.835 (19.43)	1.630 (17.89)	16.5
J01E SULFONAMIDES AND TRIMETHOPRIM							
J01EC Intermediate-acting sulfonamides	-	-	0.001 (0.01)	0.002 (0.013)	0.001 (0.014)	0.001 (0.015)	0.0
J01EE Combinations of sulfonamides and trimethoprim, including derivatives	0.016 (0.12)	0.015 (0.10)	0.018 (0.12)	0.008 (0.075)	0.003 (0.028)	0.003 (0.029)	0.1
J01F MACROLIDES, LINCOSAMIDES AND STREPTOGRAMINS							
J01FA Macrolides	0.725 (5.66)	0.809 (5.27)	0.894 (6.06)	0.776 (7.32)	0.698 (7.40)	0.729 (7.97)	6.4
J01FF Lincosamides	0.494 (3.86)	0.559 (3.65)	0.597 (4.05)	0.365 (3.45)	0.332 (3.52)	0.336 (3.69)	3.7
J01G AMINOGLYCOSIDE ANTIBACTERIALS							
J01GA Streptomycins	0.014 (0.011)	0.013 (0.078)	0.009 (0.06)	0.006 (0.050)	0.002 (0.028)	0.002 (0.029)	0.1
J01GB Other aminoglycosides	0.186 (1.44)	0.150 (0.98)	0.118 (0.80)	0.103 (0.97)	0.093 (0.99)	0.078 (0.85)	1.0
J01M QUINOLONE ANTIBACTERIALS							
J01MA Fluoroquinolones	1.219 (9.51)	1.466 (9.58)	1.366 (9.26)	1.001 (9.44)	0.907 (9.60)	0.901 (9.88)	9.5
J01X OTHER ANTIBACTERIALS							
J01XD Imidazole derivatives	0.761 (5.94)	0.846 (5.53)	0.770 (5.22)	0.683 (6.44)	0.673 (7.12)	0.658 (7.23)	6.1
J01XX Other antibacterials	0.113 (0.88)	0.143 (0.93)	0.227 (1.54)	0.173 (1.64)	0.151 (1.60)	0.153 (1.68)	1.3

Antibiotics categorized under J01AA(Tetracyclines) were not sold in primary care facilities prior to 2015.

**Table 3:** Quality indicators for antibiotic sales in outpatient in Hubei in comparison with 31 European countries (highlighted in different colours).

Year	Absolute Sales *					Relative Sales				Broad/Narrow	Seasonal Variations	
	J01_DID	J01C_DID	J01D_DID	J01F_DID	J01M_DID	J01CE_%	J01CR_%	J01DD+DE_%	J01MA_%	J01_B/N	J01_SV	J01M_SV
**2012**	21.34	8.70	6.76	2.03	2.03	5.66	2.8	18.19	9.52	2.98	6.01	1.15
**2013**	25.51	11.87	6.98	2.28	2.44	8.02	2.94	14.21	9.58	2.37	12.27	10.78
**2014**	24.57	11.04	6.87	2.48	2.28	7.82	3.39	13.8	9.26	2.63	14.39	17.24
**2015**	17.67	6.36	6.07	1.90	1.67	4.27	5.19	17.65	9.44	4.52	11.97	−15.22
**2016**	15.74	5.41	5.52	1.72	1.51	3.8	6.91	19.43	9.6	5.78	7.45	−0.88
**2017**	15.18	5.24	5.13	1.77	1.50	3.8	7.42	17.9	9.89	5.99	N/A	N/A

* Adjusted with the market share (45%) of primary care facilities in outpatient visits; 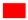 Higher than all of the compared countries; 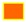 Ranked in top 25% of compared countries; 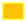 Ranked in the middle 25% of countries above median; 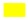 Ranked in the middle 25% of countries below median; 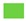 Ranked in bottom 25% of compared countries; 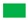 Lower than all of the compared countries.
